# Why Is COVID-19 More Severe in Patients With Diabetes? The Role of Angiotensin-Converting Enzyme 2, Endothelial Dysfunction and the Immunoinflammatory System

**DOI:** 10.3389/fcvm.2020.629933

**Published:** 2021-02-03

**Authors:** Jacob Roberts, Antonia L. Pritchard, Andrew T. Treweeke, Adriano G. Rossi, Nicole Brace, Paul Cahill, Sandra M. MacRury, Jun Wei, Ian L. Megson

**Affiliations:** ^1^Institute for Health Research and Innovation, University of the Highlands and Islands, Inverness, United Kingdom; ^2^Centre for Inflammation Research, University of Edinburgh, Edinburgh, United Kingdom; ^3^School of Biotechnology, Dublin City University, Dublin, Ireland

**Keywords:** COVID-19, SARS– CoV– 2, diabetes, endothelium, oxidative stress, angiotensin converting enzyme-2, inflammation, immune response

## Abstract

Meta-analyses have indicated that individuals with type 1 or type 2 diabetes are at increased risk of suffering a severe form of COVID-19 and have a higher mortality rate than the non-diabetic population. Patients with diabetes have chronic, low-level systemic inflammation, which results in global cellular dysfunction underlying the wide variety of symptoms associated with the disease, including an increased risk of respiratory infection. While the increased severity of COVID-19 amongst patients with diabetes is not yet fully understood, the common features associated with both diseases are dysregulated immune and inflammatory responses. An additional key player in COVID-19 is the enzyme, angiotensin-converting enzyme 2 (ACE2), which is essential for adhesion and uptake of virus into cells prior to replication. Changes to the expression of ACE2 in diabetes have been documented, but they vary across different organs and the importance of such changes on COVID-19 severity are still under investigation. This review will examine and summarise existing data on how immune and inflammatory processes interplay with the pathogenesis of COVID-19, with a particular focus on the impacts that diabetes, endothelial dysfunction and the expression dynamics of ACE2 have on the disease severity.

## Introduction

The severe acute respiratory syndrome coronavirus 2 (SARS-CoV-2) pandemic has been the focus of an unprecedented, dramatically augmented rate of scientific research since its emergence at the end of 2019. The direct and indirect health impacts of this disease have been felt by people all over the world: over 80 million people have been infected with the SARS-CoV-2 virus and the death toll has exceeded 1.75 million globally. Lockdown measures designed to curtail and contain outbreaks have led to rarely experienced increases in unemployment and many healthcare systems are being overwhelmed by severe coronavirus disease 2019 (COVID-19; the disease caused by the SARS-CoV-2 virus), lowering the rates of admission and treatment of many other health conditions. One of the most interesting and least well-understood aspects of the disease is the wide spectrum of disease severity experienced in those infected—from asymptomatic to fatal on account of respiratory or multi-organ failure. In addition, the long-term legacy of COVID-19 is, by definition, not yet fully appreciated, although worrying signs are emerging to suggest that some individuals who have survived the acute infection stage of the disease might be prone to a wide range of long-lasting health conditions (1).

A number of meta-analyses have been conducted in an effort to understand those factors that might affect the severity of COVID-19 (2–6). The outcome of these studies have identified a number of key factors that associate with critical/fatal events, most notably: gender (male, compound odds ratio (OR): 1.76), black, Asian or mixed ethnicity, BAME (OR: 1.9); age (OR: 6.06), smoking (OR: 2.04), cardiovascular disease (OR: 5.19), respiratory disease (OR: 5.15), malignancy (OR: 1.6) (2), hypertension (OR: 3.36) (7), severe mental illness (OR: 2.27) (8), and obesity (OR: 5.70) (9). Diabetes has been identified as a comorbidity associated with COVID-19 severity, with meta-analyses indicating that pre-existing diabetes is associated with a greater risk of severe COVID-19 and death (Table 1) with OR from 1.9 to 2.68 (4, 27, 32, 40, 55, 59, 64). Studies of COVID-19 patients in England found that the hazard risk for mortality was greater for patients with type 1 diabetes mellitus (T1DM: OR 2.23) than those with type 2 diabetes mellitus (T2DM: OR 1.61) (65). These findings were reinforced by a later study, which found similar mortality risks for T1DM (OR: 3.51) and T2DM (OR: 1.80) (66). In the former study, amongst patients with T2DM, hazard ratios were highest in those with poorest glycaemic control, but there was found to be an unusual U-shaped relationship with BMI and COVID-19 mortality in both types of diabetes. Association of this disease with severe COVID-19 symptoms and mortality is of particular concern on account of the spiralling prevalence of diabetes, with current estimates suggesting that >400 million people are living with diabetes worldwide and roughly half are undiagnosed (67). The rise in diabetes has been linked to rapidly escalating levels of obesity, particularly in highly populated regions of the world with known genetic pre-disposition to T2DM (e.g., South-East Asia). Until the COVID-19 pandemic, the autoimmune form of diabetes, T1DM, was considered to be relatively stable at ~1% of the population, but emerging evidence suggests that COVID-19 might be responsible for a recent rise in prevalence of T1DM (68).

**Table 1:** Meta-analyses of the mortality risk associated with pre-existing diabetes in COVID-19 patients.

**First author**	**Overall OR, RR, or RC of mortality in patients with diabetes and COVID-19 [95% CI]**	**Number of studies included in meta-analysis**	**Manuscripts included in the meta-analysis**	**References**
Mantovani et al.	OR: 2.68 [2.09–3.44]	15	(6, 10–23)	(4)
de Almeida-Pititto et al.	OR: 2.50 [1.74–3.59]	10	(6, 10, 12, 13, 17, 19, 22, 24–26)	(27)
Kumar et al.	OR: 1.90 [1.37–2.64]	9	(10, 13, 21, 25, 26, 28–31)	(32)
Huang et al.	RR: 2.12 [1.41–3.11]	10	(10, 13, 31, 33–39)	(40)
Miller et al.	RC: 1.5% [0.2–2.8]	14	(10, 39, 41–54)^*^	(55)
Hussain et al.	RR: 1.61 [1.16–2.25]	11	(10, 15, 16, 21, 25, 26, 31, 39, 56–58)	(59)
Ssentongo et al.	RR: 1.48 [1.02–2.15]	16	(6, 10, 13, 19, 21–23, 25, 26, 31, 36, 51, 60–63)	(64)

*These studies did not distinguish the type of diabetes being assessed, so the reported odds ratios (OR), risk ratios (RR), and regression coefficients (RC) are for the risk associated with any diagnosis of diabetes*.

* *These 16 studies were included in the total meta-analyses reported in this manuscript. Of these, 14 were included in the meta-analysis of diabetes associated mortality in COVID-19, however, these were not explicitly identified*.

Given the novelty of COVID-19, our understanding of the reasons for the association between severity of COVID-19 symptoms and pre-existing diabetes is poorly developed and highly complex on account of the inter-relationship of T2DM in particular with many other risk factors for severe COVID-19 symptoms (age, ethnicity, obesity, cardiovascular disease and hypertension). The aim of this review is to bring together the early evidence emerging from COVID-19 studies with that from previous, related epidemics (e.g., SARS) in an effort to understand the mechanisms by which diabetes might prime individuals to suffer more severe symptoms than their healthy counterparts. In addition, the review will examine the evidence suggesting that COVID-19 might induce a diabetes-like syndrome in some patients and identify potential mechanisms behind this.

## COVID-19

SARS-CoV-2 is transmitted primarily via respiratory droplets, with an average of 4–5 days before symptom onset (24, 69–71) and peak viral load within 5–6 days of symptom onset. COVID-19 symptoms include fever, cough, anosmia, ageusia, fatigue, myalgia, headache, sore throat, diarrhoea and dyspnoea (shortage of breath) (41–43, 72).

Severe COVID-19 cases progress to acute respiratory distress syndrome (ARDS) ~8–9 days after symptom onset (41, 42) and is characterised by pneumonia, pulmonary oedema, oxygen saturation (SpO_2_) <93%, respiratory failure requiring invasive ventilation, admission to the intensive care unit (ICU), coagulopathy, lymphopaenia, cytokine storm, viraemia, and multi-organ damage (6, 24, 42, 43, 72–74). Severe COVID-19 requires confirmation by physical and laboratory-based examinations, including SpO_2_, D-dimer assessment of fibrinolysis, inflammatory markers, leucocyte counts and computerised tomography (CT) scans (24, 75). Pulmonary oedema occurs due to excess fluid seeping out of the blood vessels in the lungs, affecting the exchange of gas (oxygen and carbon dioxide), leading to decreased SpO_2_, respiratory failure and ICU admission. Coagulopathy, including microvascular thrombosis and disseminated intravascular coagulopathy (DIC) (76, 77) and lymphopaenia could be a predictor of mortality in severe COVID-19 (78). Lung scarring and fibrosis, a hyper-inflammatory marker that suggests tissue damage due to overactive, dysregulated inflammation, occurs in survivors of severe disease. In a longitudinal study of 90 patients, CT scans showed lung abnormalities soon after the onset of symptoms. Ground-glass opacity was the most common sign, with an increase in a mixed pattern phenotype observed 12–17 days following the onset of symptoms (79). Another clinical report revealed that 58.3% of severe cases had bilateral patchy opacities compared to 30.4% of non-severe cases, suggesting the presence of a radiographic marker in severe COVID-19 (24). Lung autopsies of deceased COVID-19 patients demonstrated that neutrophil extracellular traps (NETs) were likely to be involved in inflammation-associated lung damage, thrombosis and fibrosis (80). Lung autopsies also showed type-2 pneumocyte hyperplasia in all cases examined (81), potentially suggesting a compensation for the loss of angiotensin converting enzyme 2 (ACE2) expressing cells in the lung. Severe infections have been associated with a sustained high viral load in the upper airways (82, 83) and patients who have died from severe COVID-19 showed SARS-CoV-2 virions can be detected in almost all tissues, including the brain (84, 85).

A substantial proportion of people infected with SARS-CoV-2 are asymptomatic. However, an accurate rate of asymptomatic infection of SARS-CoV-2 is difficult to ascertain due to the limitations of testing policies. Large-scale longitudinal testing of populations is required to provide a more precise picture of the number and demographics of asymptomatic carriers; computational modelling has predicted the number of infections to be 3 to 20 times higher than the number of confirmed cases (86). Asymptomatic infections could be due to the efficacy of the host immune responses, low viral load, cross-reactivity of existing immune effectors, or prior infection with/pre-existing immunity to other related human coronaviruses, conferring cross-immunity against SARS-CoV-2 (87–89) and resulting in a longer period of detection of viral RNA in the upper respiratory tracts of symptomatic patients (89).

To date, studies in COVID-19 hotspots have revealed that <20% of infections are classed as “severe,” while the remaining 80% are asymptomatic, mild, or moderate (24, 90). While there has been little evidence that demographic factors and co-morbidities impact on the risk of SARS-CoV-2 infection, it is clear they influence the severity of symptoms of COVID-19. That there is such a wide range of symptoms was initially perplexing; however, the associations with age, ethnicity and co-morbidities have provided vital clues as to why some individuals develop specific severe symptoms or succumb to their infection.

### SARS-CoV-2

**Figure 1:**
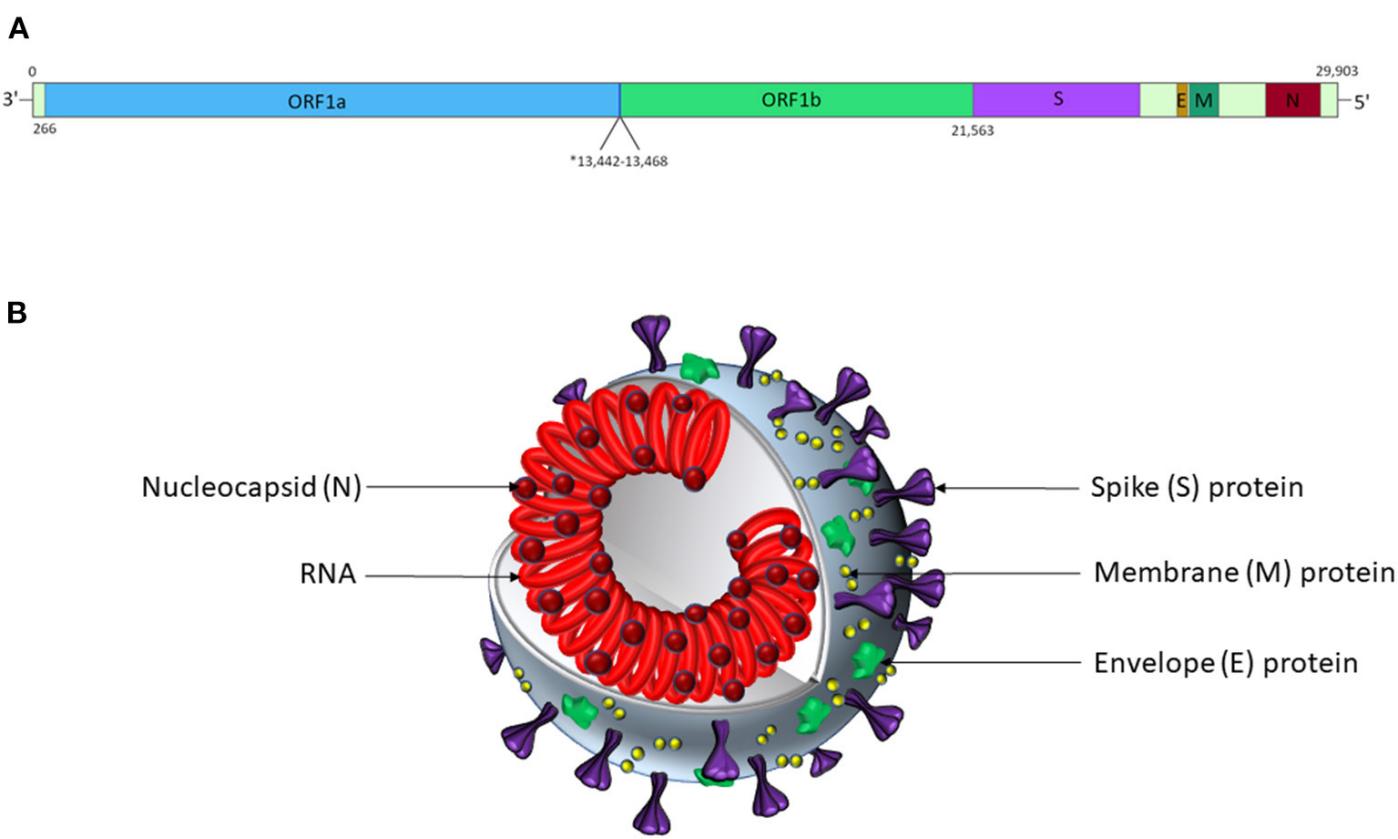
SARS-CoV-2 genomic and virion structures. **(A)** Genome schematic of SARS-CoV-2. The asterisk marks the overlapping reading frames of ORF1a and ORF1b, which encode a variety of non-structural proteins, including helicases, proteases, and an RNA-dependent RNA polymerase. The S protein is encoded by nt. 21,563-25,384; the E protein is encoded by nt. 26,245-26472; the M protein is encoded by nt. 26,523-27,191; and the N protein is encoded by nt. 28,274-29,533. Accessory proteins not shown. Adapted from Alanagreh et al. (91). **(B)** Schematic representation of the SARS-CoV-2 virion structure. This figure is not to scale, and relative abundances of the proteins shown are arbitrary.

SARS-CoV-2 is a positive-sense, single-stranded RNA virus in the *Nidovirales* order and *Betacoronavirinae* genus (Wuhan-Hu-1 isolate reference genome NCBI ID: 86693; transcript ID: NC_045512.2; Figure 1) (92, 93). It is one of several coronaviruses that infect the human respiratory system, including SARS-CoV-1 (causing severe acute respiratory syndrome, SARS) (92, 94), MERS-CoV (causing Middle East respiratory syndrome, MERS) (92, 95), HCoV-229E, HCoV-HKU1, HCoV-NL63, and HCoV-OC43 (92). The virion encodes proteins required for viral replication (Figure 1), including the RNA-dependent RNA polymerase (RdRp) and structural proteins, including spike (S), membrane (M), nucleocapsid (N) and envelope (E) (96), which are likely to function similarly to those described in related coronaviruses.

### The S Protein and Cellular Entry via ACE2

**Figure 2:**
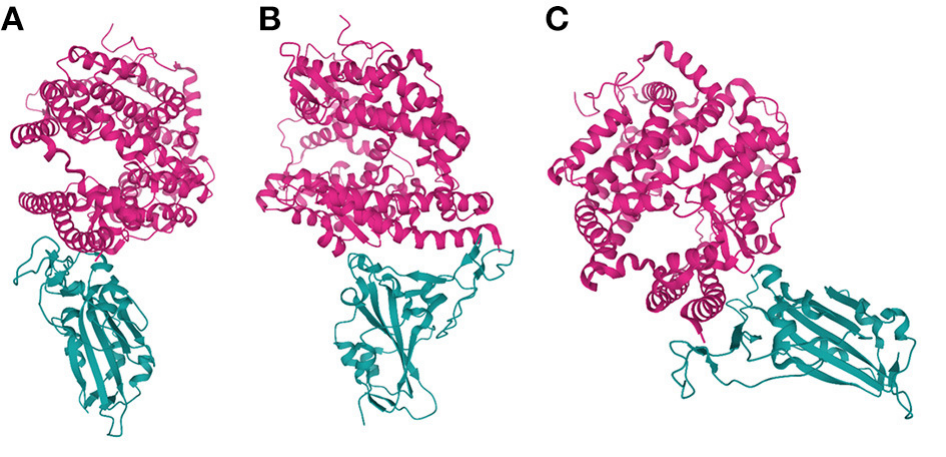
SARS-CoV-2 S interfacing with ACE2. Various orientations of an X-ray crystallography-derived cartoon representation of the Spike protein S1 subunit of SARS-CoV-2 (cyan) bound to an extracellular portion of ACE2 (residues 1-597; pink). **(B)** Is a 90° anticlockwise rotation, along the z-axis, of **(A)**, while **(C)** is angled to highlight the shape of the binding interface between ACE2 and SARS-CoV-2 S, ~90° clockwise along the z-axis, and ~45° counter-clockwise along the x-axis, relative to A. Figure was created using *Mol. PDB ID: 6M0J, Crystal Structure of SARS-CoV-2 spike receptor-binding domain bound with ACE2 (97, 98).

The S protein of SARS-CoV-2 is a homotrimer and, like that of SARS-CoV-1, is a class I fusion protein [13] that recognises the host cell receptor, ACE2 as a key step in the viral internalisation process (Figure 2). ACE2 (Xp22.2) is a zinc metalloprotease with homology to both ACE and collectrin (CLTRN) (99, 100). X-ray crystallography has confirmed that the SARS-CoV-2 binding interface is similar to that of the SARS-CoV-1 S protein (97) and *in vitro* experiments have shown that SARS-CoV-2 internalisation requires ACE2 (101, 102).

Upon binding to ACE2, the conformation of the S protein changes to initiate the membrane fusion of virus particles and host cell, required for internalisation of the viral genome. The S1 subunit of the protein contains the receptor binding domain (RBD), which interfaces with the N-terminus of ACE2 on target host cells. The S2 subunit contains two distinct regions of heptad repeats, as well as a fusion domain, all of which are essential for viral entry into host cells (103). Following viral binding to ACE2, the heptad repeats of S2 associate to each other, forming a six-helical bundle (6HB) complex (104–106). In SARS-CoV-1, the formation of 6HB exposes the fusion domain of the S2 subunit, which is hydrophobic and embeds into the host cell membrane, initiating fusion (105).

The S protein exists in either “standing up” or “lying down” conformations prior to receptor binding. When standing up, the RBD of the S protein is more exposed, allowing for higher affinity binding to ACE2, but also presenting a highly immunogenic target (107–109). There is a 4 amino acid insertion seen in the SARS-CoV-2 S protein that is not present in SARS-CoV-1, which creates a furin cleavage site at the junction between the S1 and S2 subunits (103). *In vitro*, pre-treatment with furin enhances viral entry into ACE2 expressing cell lines, which is hypothesised to cause a transition from lying down to the standing up conformation (109).

The entry of SARS-CoV-2 into cells via ACE2 also relies on transmembrane serine protease 2 (TMPRSS2), which primes the S protein through proteolytic cleavage, altering its conformation in a manner that facilitates membrane fusion (109).

## Normal Physiological Role of ACE2

**Figure 3:**
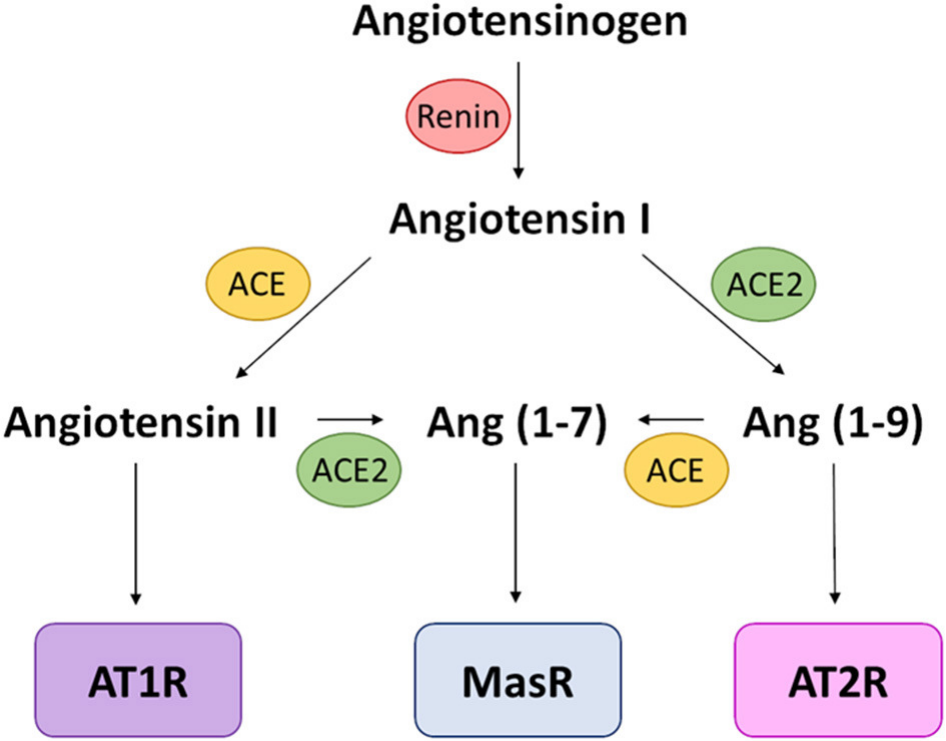
RAAS schematic. Simplified schematic focusing on the activity of ACE and ACE2 and the resulting receptor activation. The vasoconstrictive activity of this pathway is mediated by AT_1_R activation, which also causes increased oxidative stress; MasR and AT_2_R activation leads to vasodilation and a reduction in oxidative stress. Receptors are in boxes; enzymes that processes signalling peptides are in ovals.

The main physiological role of ACE2 centres on its metalloprotease activity, processing multiple proteins involved in the renin-angiotensin-aldosterone-system (RAAS; Figure 3). Specifically, ACE2 converts the vasoconstrictor agents angiotensin-I into angiotensin-(1–9) and angiotensin-II into angiotensin-(1–7), both products being vasodilatory (110, 111). The action of ACE2 therefore shifts balance of vascular control in favour of vasodilation (112). A receptor for angiotensin-II, AT_1_R, is also present on macrophages, dendritic cells, T-cells, mesangial cells and vascular smooth muscle cells, meaning that ACE2 processing of angiotensin-II also influences function of these cells (113). Therefore, in addition to its role in SARS-CoV-2 cellular entry, ACE2 has normal physiological functions that directly link to COVID-19 features in the lung, including inflammation, oxidative stress and fibrosis (113).

In addition to its role in the RAAS, ACE2 inactivates the vasodilator, bradykinin. It is important to note that there is complex interplay between RAAS and the bradykinin system, with multiple feedback loops and intersections. The importance of bradykinin in the COVID-19 story has gained credence recently on account of an unbiased *in silico* approach to understanding the underlying reason for differential severity in COVID-19 symptoms (114). Bradykinin ligates specific G-protein-coupled receptors to mediate vasodilatation via endothelial nitric oxide (NO) and prostacyclin (PGI_2_), which drive vascular permeability and local oedema. Leaky capillaries in the lungs of COVID-19 patients would not only compromise breathing on account of oedema, but would also increase trafficking of inflammatory cells between the blood and lung tissue, together with the inflammatory mediators they convey.

Following the identification of ACE2 as the target of the S protein of SARS-CoV-1 [reviewed by (115)], ACE2 was shown to be expressed by lung alveolar epithelial cells, small intestine enterocytes, vascular endothelial cells and arterial smooth muscle cells derived from the brain, lungs, kidneys, large intestine and small intestine, using immunohistochemistry (116). ACE2 is also highly expressed by nasal epithelial cells (83), which is pertinent in the pathogenesis of cellular infection in the upper airways. Single-cell RNA sequencing has shown ACE2 mRNA expression in a range of cells: type II alveolar cells, myocardial cells, kidney proximal tubule cells, urothelial bladder cells, ileal epithelial cells and oesophageal epithelial cells, though this study did not examine protein levels (117).

## Soluble ACE2

**Figure 4:**
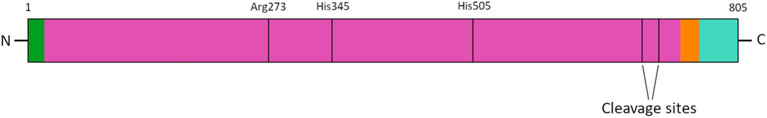
ACE2 protein schematic. An annotated schematic of the primary structure of ACE2. Arg273 is essential for substrate binding, by facilitating the formation of a salt-bridge. His345 and His505 assist with stabilising the substrates transition state during catalysis (118). The cleavage sites for the *in vivo* production of sACE2 are Arg697 and Lys716.

ACE2 also exists in a soluble form (sACE2) *in vivo*, generated by the activity of TMPRSS2 and of a disintegrin and metalloproteinase domain 17 enzyme (ADAM-17; also known as tumour necrosis factor-alpha converting enzyme, TACE). These enzymes cleave ACE2 at p.R697 and p.K716 [Figure 4; (119, 120)]. Increased sACE2 activity has been proposed as a possible biomarker, predictive of poor patient outcome and heart failure following myocardial infarction or heart transplant (121). Whilst it has not yet been demonstrated that sACE2 is able to convert vasoconstrictor angiotensins to their vasodilator counterparts *in vivo*, assays using fluorogenic substrates show that sACE2 can hydrolyse angiotensin-I and angiotensin-II analogues (122). In addition, the initial characterisation of ACE2 that utilised a truncated, soluble version of the protein, indicated the production of angiotensin-(1–9) and angiotensin-(1–7), by mass spectrometry (123).

It has been suggested that cleavage of membrane-bound ACE2 to generate sACE2 may serve as a feedback mechanism in the RAAS. Evidence for this was presented in a mouse study, where continuous subcutaneous angiotensin-II release, via osmotic minipump, decreased myocardial membrane-bound ACE2 protein levels and increased the levels of sACE2 activity in plasma (122). This finding was reinforced *in vitro* in the human hepatocellular carcinoma cell line (HuH-7), with angiotensin-II exposure resulting in increased levels of ACE2 activity and sACE2 in supernatant (122).

The soluble portion of ACE2 comprises nearly the entirety of the protein's extracellular domain and could therefore plausibly act as a ligand for the SARS-CoV-2 S proteins. However, the conformation of the truncated protein has not been confirmed. While no evidence has been presented for how frequently an interaction between virions and endogenous sACE2 happens in COVID-19 patients, a case study showed that intravenous infusion of human recombinant soluble ACE2 (hrsACE2) could successfully treat severe COVID-19 (124); the putative mechanism behind this treatment may involve preventing SARS-CoV-2 from entering ACE2-expressing cells for replication and thereby-reducing angiotensin-II levels in the circulation. Moreover, the use of sACE2 as “bait” to sequester SARS-CoV-2 to potentially slow/halt the spread of infection in patients with viraemia is currently being investigated (122, 125).

## Overview of Immune Response to SARS-CoV-2 Infection

SARS-CoV-2 initially infects cells of the upper airways. During the initial incubation phase, SARS-CoV-2 replicates in infected cells without detectably triggering the innate immune response, leading to initially high viral loads (126). During the robust phase of SARS-CoV-2 cellular infection, or uptake of virions by antigen-presenting cells (APC), toll-like receptors (TLR), including TLR3, TLR7, and TLR8 (127) are stimulated, producing a rapid innate immune response (128–130). This includes initiation of a signalling cascade that promotes the production of innate interferons (IFN-α, IFN-β, IFN-λ) via interferon regulatory factors (such as IRF1 and IRF7) (131) and pro-inflammatory mediators via NF-κB (132). Stimulator of interferon genes (STING) also performs a key role in the innate immune system's defence against viral infection [reviewed by (133, 134)]. The signalling of STING via NF-κB and IRF3 was initially shown to be stimulated by the presence of non-CpG intracellular DNA but there is evidence for its activity in response to viral RNA as well [reviewed by (135)]. Excessive STING activity induces pyroptosis in monocytes and macrophages, as well as elevated tissue factor (CD142) levels (136), both of which occur in severe COVID-19 patients (73, 137, 138). The overexpression of angiotensin-II activates the STING pathway in murine myocardial cells (139), which may indicate a route by which STING is overstimulated in COVID-19 patients with cardiovascular disease and T2DM. These rapid responses stimulate different effects, depending on the cell type in which they occur [reviewed in (140)].

Cross-reactive T-cell responses directed against either the SARS-CoV-2 spike or membrane proteins were present in healthy individuals who donated blood before the pandemic; in ~50% of CD8^+^ T-cells and ~30% of CD4^+^ T-cells (88, 141, 142). The majority of individuals who have had confirmed SARS-CoV-2 infection had a robust T-cell (88, 141, 142) and B-cell response, with detection of IgA, IgM, and IgG antibodies against the virus, which lasted at least 6 weeks (143–145). However, a long-lasting B-cell response was less common in those with mild COVID-19 (146). The adaptive immune response is initiated by intermediatory cells activated by innate effectors, such as dendritic cells that display antigen to CD8^+^, CD4^+^ helper T-cells (proinflammatory T_h_1 and T_h_17 cells and suppressive Th2 cells) and CD4^+^ T_reg_. B-cell responses can be either T_h_ cell dependent or independent. Cytokines secreted during T-cell dependent activation encourage B-cell proliferation and isotype switching to maintain germinal centre size and longevity. Notable differences in these responses have been observed in patients with mild compared to severe COVID-19 [review by (147)].

### Immune Response Differences in Mild vs. Severe COVID-19

A range of immune markers are markedly dysregulated in patients with severe complications from COVID-19 compared to those with mild/moderate infection, which provides clues as to the reason some people have a severe response to SARS-CoV-2 infection (Table 2) (10, 42, 126, 148–155).

**Table 2:** Immune mediators dysregulated in severe COVID-19.

**Immune mediator**	**Cells producing mediator**	**Alteration in the circulation of severe COVID-19 patients**	**References**
IFN-α, IFN-β, IFN-λ, IFN-γ	T-cells, B-cells, Plasmacytoid dendritic cells, NK-cells, macrophages, endothelial cells, fibroblasts	Increased	(42, 148–150)
		Lower	(126, 151)
CCL2 (MCP-1)	Monocytes, macrophages, dendritic cells, fibroblasts	Increased	(42, 126, 151, 152)
CCL5 (RANTES)	T_H_1 cells, CD8^+^ T-cells, macrophages mucosal epithelial cells, NK cells	Increased	(152)
CCL7 (MCP-3)	Dendritic cells, monocytes, macrophages	Increased	(152, 153)
IP-10 (CXCL10)	Alveolar epithelial cells, monocytes, fibroblasts	Increased	(42, 153, 154)
MIP-1a	Macrophages and monocytes	Increased	(42, 152, 154)
G-CSF	Endothelial cells, fibroblasts, monocytes	Increased	(42, 148, 152, 153)
TNF-α	Macrophages, monocytes; FSC^hi^ monocytes	Increased	(42, 151, 152, 155–157)
IL-1β	Endothelial cells, macrophages	Increased	(152, 153)
		Low/normal	(151, 156)
IL-1RA	Macrophages, T_H_1 cells, NK cells	Increased	(126, 152)
IL-2	CD8^+^ T-cells, CD4^+^ T-cells, smooth muscle cells	Increased	(42, 148, 152)
IL-2R	Macrophages, monocytes, neutrophils, fibroblasts, Sertoli cells, microglia	Increased	(153, 155)
IL-6	CD8^+^ T-cells, T_H_1 cells; FSC^hi^ monocytes	Increased	(126, 148, 151, 153–157)
IL-7	Dendritic cells, epithelial cells, microglia, hepatocytes, keratinocytes, stromal cells	Increased	(42, 148, 152)
IL-8	Endothelial cells	Increased	(148, 152, 155, 156)
IL-10	FSC^hi^ monocytes	Increased	(42, 148, 151, 152, 155, 157)
IL-17	T_H_17 T-cells, endothelial cells	Increased	(152, 158)

In patients with severe COVID-19, there is robust production of pro-inflammatory cytokines and chemokines, with a limited production of IFN-α, IFN-β, and IFN-λ (Table 2), suggesting an effective activation of NF-κB but not of IRF7 (126), proposed to result in an imbalance between the pro-inflammatory vs. pro-repair functions of airway macrophages (150). One of the features of severe COVID-19 that has been extensively described is a “cytokine storm,” which can lead to high risk of vascular hyperpermeability, multiorgan failure, and death [reviewed in (159–161)]. IL-6 is a key biomarker for COVID-19-related cytokine storms (162) and has shown an inverse correlation with impaired immunity (159, 163); T-cell numbers were negatively correlated to the concentrations of serum IL-6, IL-10, and TNF-α (164). Severe COVID-19 is consequently associated with impairment of T-cells, manifesting as lymphopenia and functional exhaustion of CD4^+^ (T_h_ and T_reg_) and CD8^+^ T-cells; it is likely that the preceding innate immune dysregulation influences these observations (155, 163–165).

The B-cell response is also likely dysregulated, although reports are inconsistent, confounded by methodological issues surrounding the detection of antigen-specific immunoglobulins (166, 167) and whether systemic (circulatory) or local (particularly mucosal) responses were assessed (168). For example, enhanced secretory mucosal IgA responses, detected in the circulation in severe COVID-19, were hypothesised to confer damaging effects via induction of inflammatory cytokines (169–171); although IgA levels in saliva from COVID-19 patients showed only a moderate correlation with COVID-19 severity (172) and other studies found no difference with disease severity (155). Circulating IgG levels were higher in patients with severe COVID-19, which has been hypothesised to promote macrophage hyper-inflammatory responses (173); although the effect of viral load on the secretion of antibodies has not been ruled out.

### Immune Dysregulation in Diabetes

There are chronic, persistent immune dysregulation features present in T1DM and T2DM, which are associated with disease-related pathology. In both T1DM and T2DM, there are notable changes in cytokine expression and phagocytic activity, such as suppressed chemokine responses, higher levels of pro-inflammatory mediators and lower rates of phagocytosis by neutrophils and macrophages necessary for uptake of infectious antigen (174–179).

The β-islet destruction that is a feature of T1DM is a consequence of the action of CD4^+^ and CD8^+^ T-cells with specificity for islet autoantigens (180), and local macrophages secreting pro-inflammatory cytokines (181); autoreactive antibodies are a secondary consequence to this. Individuals with T1DM do not have differences in the number of T_reg_ cells compared to healthy individuals (180), but do have defects in T_reg_ activation, which persist throughout their lifetime (182). Furthermore, the function of existing T_reg_ is dysregulated, whereby their suppressive capabilities are reduced, leading to a sustained increase in pro-inflammatory cytokines (180).

In T2DM, the major immune feature is an imbalance in the T_h_17 and T_reg_ cells, which reflects a loss in T-cell homeostasis and is a major contributor to inflammation and tissue specific immunity (183). T_h_17 cells have unique glycolysis and lipogenesis metabolic profiles that drives differentiation and cytokine production (184). The significant alterations in lipolysis and lipogenesis in patients with T2DM impacts T_h_17 function and suggests that despite the normalisation of blood glucose levels in T2DM, this might not be sufficient to reverse obesity-associated T-cell inflammation (185).

## What Features in Diabetes Might Exacerbate or Prolong COVID-19 Symptoms?

### Impact of Hyperglycaemia

The association of diabetes with severe symptoms in COVID-19 is linked to glucose control, with little apparent dissociation of the effect between patients with T1DM and T2DM (65). Hyperglycaemia impairs host defences, including granulocyte and macrophage function and lymphopaenia, as further discussed below. People with diabetes are at increased risk of a wide range of infections, with death from infections substantially higher than in matched controls, particularly amongst patients with T1DM [OR 7.72; (186)]. HbA1C is a marker of glycaemic control that is closely associated with risk of infection, hospitalisation, and mortality (187). While these data provide interesting insights in terms of susceptibility to infection, severity, and mortality, they provide little information regarding the mechanism underpinning the association. An interesting additional observation, however, is that fasting blood glucose (i.e., acute, transient measure of glucose regulation) at hospital admission has been found to be an independent predictor of COVID-19 mortality in patients without pre-existing diabetes (188), which lends weight to the hypothesis that blood glucose itself is a key mediator of severe symptoms in COVID-19.

### Dysregulation of RAAS in COVID-19: Intersection With Diabetes-Mediated RAAS Dysfunction?

Perhaps the most controversial area in the COVID-19 story to date is the potential role of vasoconstrictor/pro-inflammatory angiotensin-II balance with vasodilator/anti-inflammatory angiotensin-(1–7) and -(1–9) in the pathophysiology of the disease and the development of severe and complex symptoms (Figure 3). Early reports during the pandemic suggested that angiotensin-II was found to be increased (3-fold) in patients with COVID-19 (189), although this finding has yet to be replicated (190). The initial advice around ACE inhibitors was mixed (191, 192) and in the face of this controversy, the early advice was not to prescribe ACE inhibitors or AT_1_R antagonists, but equally not to withdraw them from patients prescribed these drugs for pre-existing conditions (193–195). The latest study relating to ACE inhibitors suggest some benefit in terms of COVID-19 disease as a whole, if not reduced risk of ICU care (196) without increased risk of death (197, 198); however, there was also a complex interaction effect with ethnicity in that benefits were less apparent or absent in Black African groups in particular.

**Figure 5:**
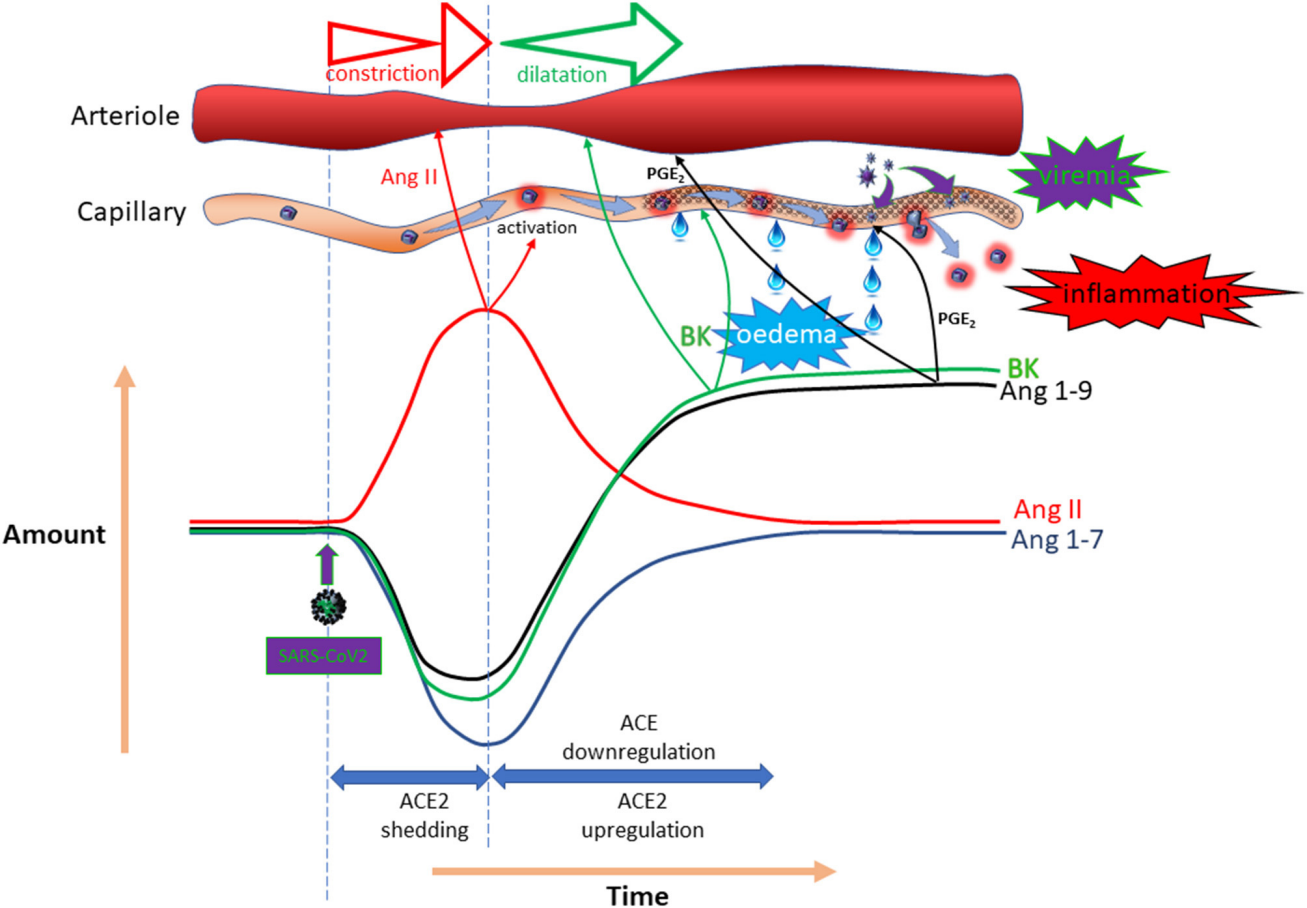
Potential temporal impact of COVID-19 on the RAAS. Hypothetical impact of SARS-CoV-2 on local expression of ACE/ACE-2 enzymes and the possible outcome in terms of angiotensin-II, angiotensin 1–7, angiotensin 1–9 and bradykinin (BK) concentrations, as well as the vascular impact.

The primary reason for the conflicting suggestions and outcomes is the complex counter-regulatory mechanism that applies in this system. While loss of ACE2 to SARS-CoV-2 seems inevitable, expression of this enzyme is often found to be substantially up-regulated on account of a counter-regulatory mechanism mediated by ACE/angiotensin-II (199). Simultaneously, there appears to be down-regulation of ACE (200). In addition, an *in silico* study using worldwide databases suggested that there was a significant reduction in ACE2 expression in patients with diabetes (201). The ultimate impact of SARS-CoV-2 on RAAS mediators is likely to fluctuate with time and to be highly dependent on viral load. Because of the differential importance of ACE2 in metabolism of the various mediators, the response profiles will be distinct. Figure 5 provides a hypothetical response profile for some for the key mediators impacted by SARS-CoV-2, illustrating the importance of the time-course of the various responses on the pathophysiological outcome at any given time. In the event that a course of events similar to that illustrated is experienced, it is clear that both the amplitude of the impact (dictated by viral load) and the time-course and effectiveness of the various counter-regulatory events will have a substantial bearing on the nature and extent of the symptoms experienced. Superimposition of pre-existing ACE inhibition or AT_1_R antagonism on proceedings adds another layer of complexity, perhaps explaining the variety of outcomes and opinions relating to this particular topic. What is clear is that “one size fits all” does not apply in this regard.

**Figure 6:**
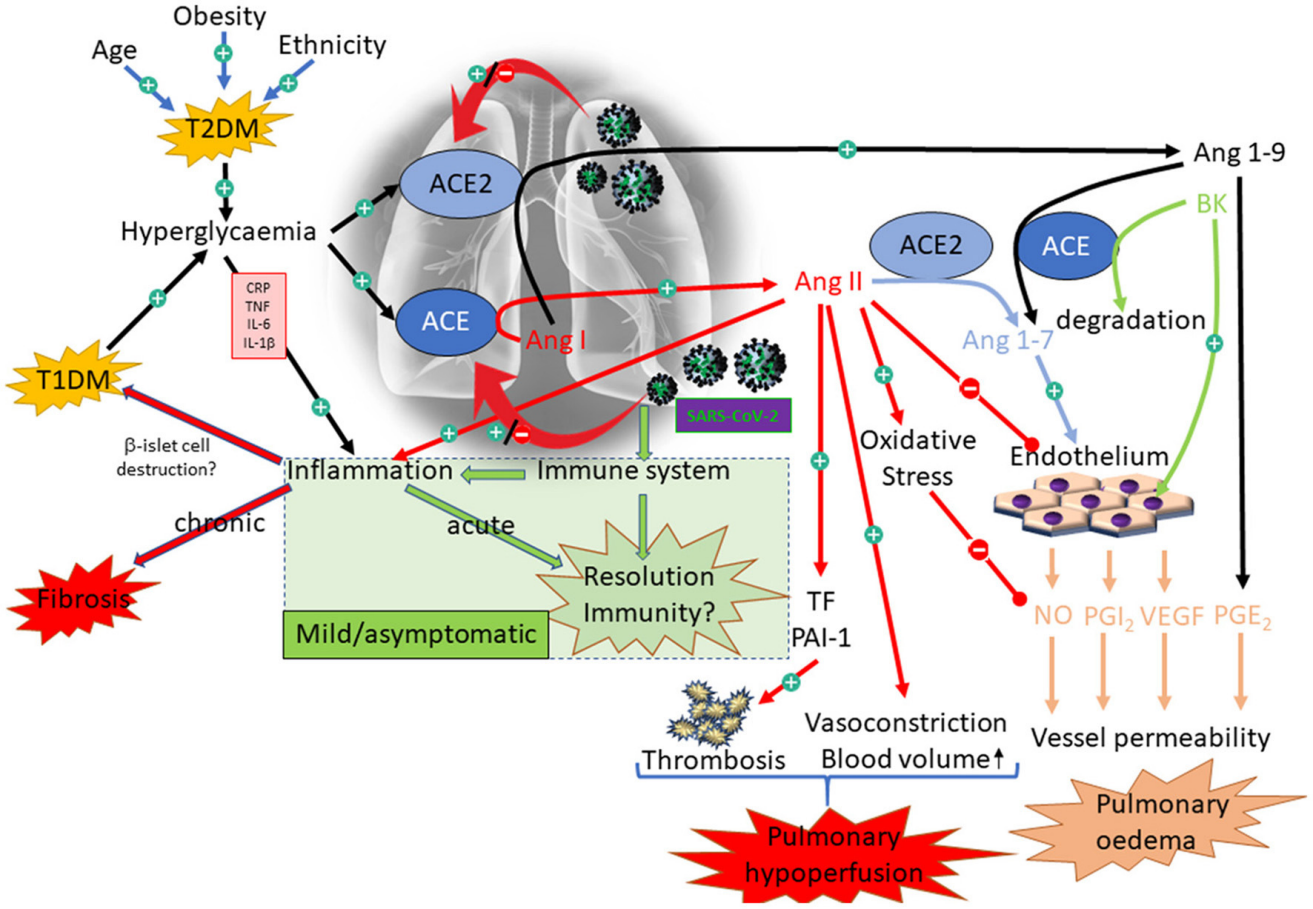
Interaction between diabetes and COVID-19 with respect to the RAAS. Illustration of the complex interactions associated with diabetes and COVID-19. The green box illustrates the appropriate response to SARS-CoV-2, resulting in only mild symptoms. The alternative pathways illustrate some of the dysregulation that might be primed by diabetes, resulting in acute (pulmonary hypoperfusion and oedema) and chronic (fibrosis) outcomes.

There is consensus that dysregulation of the RAAS is a feature of diabetes and represents a valid target for drug intervention, particularly with respect to renal complications associated with the disease [e.g., nephropathy; reviewed here: (202)]. The precise nature of the dysregulation, however, is much less clear, with claims and counter-claims as to the relative expression of ACE and ACE2 in diabetes; certainly evidence from animal models appears to point to down-regulation of ACE2 in the kidney (203). Perhaps the most relevant evidence, however, is derived from a genome wide association study (GWAS) on markers associated with increased pulmonary ACE2 expression (204). This study notably identified association with polymorphisms previously linked to T2DM; although the association did not withstand correction for multiple testing (at a false discovery rate of <0.05), so should be viewed with caution. The association was weaker for T1DM (204). Taking all these data together, it is conceivable that ACE2 expression is differentially affected in different tissues within an individual with diabetes and that the pattern of effect on ACE2 varies from person to person. This concept is supported by a study from an animal model in which the expression and activity of ACE and ACE2 were measured in various tissues from a non-obese, diabetic mouse model; the pattern of expression of ACE and ACE2 varied dramatically across serum and various tissues, including lung, where ACE2 expression was increased, but to a lesser extent than ACE, resulting in an overall decrease in ACE2:ACE in this tissue. The reverse was true in serum (205). This variance is critical in understanding the impact of COVID-19 at tissue/organ level with respect to susceptibility to COVID-19: higher ACE2 expression in the lungs of patients with diabetes might correspond to increased susceptibility to binding and subsequent replication of SARS-CoV-2, which in turn could drive a further up-regulation of ACE2, resulting in bradykinin and angiotensin 1–9-mediated pulmonary oedema [hypothesised here: (206)]. Decreased renal ACE2 in the same individual might, however, offer some protection from viral infection associated with viraemia. Figure 6 is a hypothetical illustration of the complex interactions that might accrue in diabetes on account of modulation of ACE/ACE2 expression by COVID-19 and diabetes.

## Key Factors in the Interplay Between COVID-19 and Diabetes

While the RAAS has been the focus of much attention in COVID-19 on account of the key role of the ACE2 receptor, there are a number of other critical elements associated with viral infection and host response that may be influenced by, but are not entirely dependent upon, changes in RAAS activity. Each of these elements are also affected by both types of diabetes, with the potential to increase susceptibility to severe symptoms in COVID-19.

### Do the Dysregulated Immune Features Associated With Diabetes Lead to a Severe COVID-19 Response?

As previously described, T1DM and T2DM are associated with distinct, chronic immune profiles. Pre-pandemic, it was already established that patients with diabetes had a greater risk of lower respiratory tract infections (207) and more severe outcomes to respiratory infections [e.g., MERS (208), influenza (209), and bacterial (207)]. There are notable confounders to the poor outcomes to respiratory viruses (including COVID-19) associated with diabetes, particularly increased age and male sex, which are also factors previously linked to poor immune response to other respiratory viruses (210–213), risk of T2DM (214) and poorer outcomes to COVID-19 (215). Lower respiratory tract infections occur when the immune response in the upper airways fails to contain the viral spread, which is a key feature of severe COVID-19. Therefore, it is plausible the immune dysregulation in T1DM and T2DM is a contributor to the less favourable outcomes with SARS-CoV-2 infection. Of course, it is an over-simplification to limit diabetes to T1DM and T2DM: there are significant patient subsets within these populations, at least some of which display significant differences in B-lymphocyte populations (216). It is not yet known whether susceptibility to severe COVID-19 symptoms align with certain diabetes sub-populations on account of their B-cell function or whether these subtleties are overwhelmed by other contributory factors like age, glycaemic control, ethnic origin or BMI.

Patients with T1DM have a dysregulated T_reg_ response and patients with T2DM have an aberrantly active T_h_17 response, both of which lead to a sustained increase in pro-inflammatory cytokines (180, 182, 183, 185). The increased pro-inflammatory cytokine profile already present in T1DM and T2DM could therefore skew the immune response to SARS-CoV-2 infection toward an inflammatory response, increasing the likelihood of severe COVID-19, with associated cytokine storm, tissue damage, and respiratory failure. Given the propensity for SARS-CoV-2 to require a higher viral load to trigger detection by the immune system even in healthy individuals, the capacity to raise an acute immune response might be further compromised in patients with diabetes, resulting in a viral load that rapidly overwhelms the immune response capacity. Patients hospitalised with diabetes and COVID-19 who had a more controlled blood glucose concentration had lower incidences of lymphopaenia (30.5 vs. 49.6%) and neutrophilia (10.7 vs. 19.4%) than patients with a high (>7.5 mmol/L) blood glucose concentration, indicating that good glycaemic control is also important in maintaining a balanced immune system (11, 215).

Several reports demonstrate that persistent inflammation is associated with a compensatory anti-inflammatory response that could prevent excessive tissue damage by increasing immunosuppressive activity (217–219). The anti-inflammatory responses include the induction of myeloid-biassed haematopoietic stem cell differentiation, increased expansion of T_reg_ cells and increased expression of anti-inflammatory cytokines such as IL-10 and TGF-β (219). The activation of immunosuppressive cells suppresses the functions of both innate and adaptive immunities. Accordingly, T2DM-related immunosuppression may facilitate SARS-CoV-2 replication in ACE2-expressing cells and the development of damage-associated molecular patterns (DAMP)-promoted cytokine storms in these patients.

In summary, from the gathered evidence of altered immune mediators in patients with T1DM and T2DM without SARS-CoV-2 infection (174–180, 182, 183, 185) and more severe outcomes during respiratory infections, including SARS-CoV-2, (6, 10, 41–43, 72, 73, 155, 186, 187, 207–213), it is highly likely that at least part of the poor outcome in patients with diabetes to COVID-19 is due to dysregulated, abnormal immune responses.

### Do Critical Changes in the Immune Response and ACE2 Expression With Age Predispose to Severe COVID-19?

People over 60 years of age have a weaker immune response to respiratory infection compared to younger individuals (210–213) and coordinated immune responses specifically to SARS-CoV-2 are disrupted in individuals aged over 65 years old (220), indicating a reason why elderly people have high risk of developing severe COVID-19 after infection with SARS-CoV-2 virus (221). Individuals with impaired immune function cannot effectively clear SARS-CoV-2 from the upper respiratory tract and as a result, the virus can quickly disseminate to the lung with rapid replication in ACE2-expressing cells. Older patients with COVID-19 can reduce their viral titres, only to rapidly descend into a state of shock due to hyperactivation of the immune system, known as a cytokine storm. One mechanism by which this might occur is via virus-injured cells releasing the DAMPs that can promote hyperinflammation (222, 223).

Another key aspect associated with age that might influence severity of COVID-19 symptoms is ACE2 receptor expression. It is known that ACE2 receptor expression in epithelial and endothelial cells in rat lungs fall substantially in older individuals and is lower overall in male compared to female animals. This concept is interrogated in a recent viewpoint article, which postulates that a reduced ACE2 expression profile at onset of COVID-19 results in an exaggerated inflammatory response on account of the acute additional loss of ACE2 receptors in the face of the virus (224).

### Do the Metabolic Features of T2DM, Obesity and Cardiovascular Disease Play a Role in Development of Severe COVID-19?

**Figure 7:**
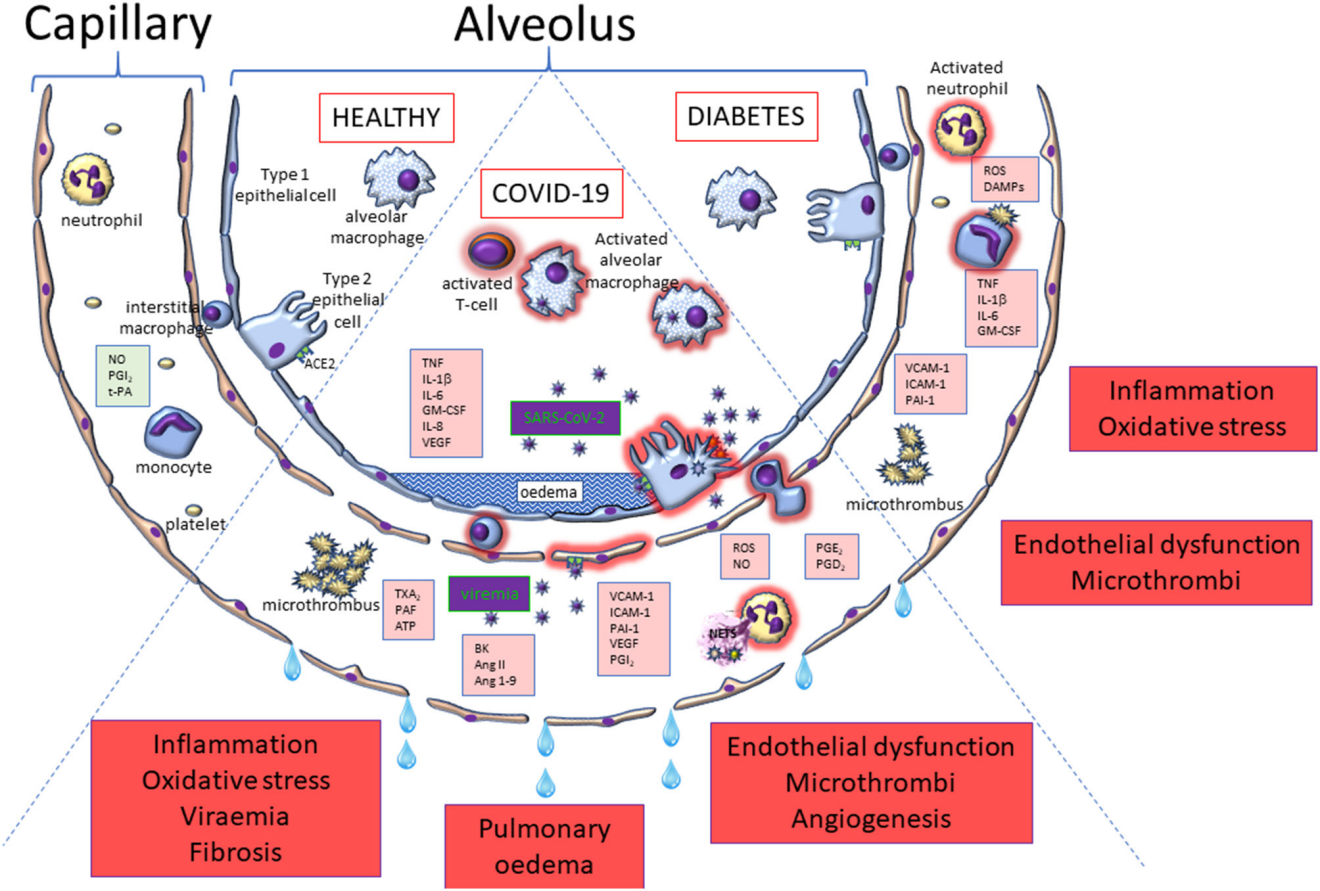
Alveolar and capillary pathophysiology relevant to COVID-19 and diabetes compared to the healthy scenario. This figure highlights some of the key cells that become activated in COVID-19 (middle segment) and diabetes (right segment) that have the potential to drive dysfunction in endothelial (diabetes and COVID-19) and alveolar epithelial (COVID-19 only) cells. The pathophysiological consequences are highlighted in the red boxes.

While the mechanism behind severe COVID-19 is unknown, individuals with conditions associated with metabolic disorders such as obesity, diabetes and cardiovascular disease have a higher risk of developing the cytokine storm and coagulopathy that play crucial roles in progression to a critical clinical condition (225, 226). The risk of in-hospital deaths with COVID-19 was significantly higher in patients with diabetes than those without diabetes, with hazard ratio of 2.36 (227). Because metabolic disorders are associated with NET dysfunction (222, 228, 229), an increase in activated neutrophils and NET formation may contribute to severe COVID-19 in patients with T2DM and obesity. Increased neutrophil-to-lymphocyte ratios have been identified as an early indicator of SARS-CoV-2 infection, predicting severe COVID-19, cytokine storm and poor clinical outcomes (41, 230). A recent study revealed that NETs triggered by SARS-CoV-2 were likely to depend on ACE2, virus replication, serine protease, and protein arginine deiminase 4 (231). Furthermore, it has been reported that patients with T2DM had an elevated NET release after infection, not only with SARS-CoV-2 (232), but also with other invading microbes (233). This suggests that hyperglycaemia may be a key contributor to the formation of NETs in COVID-19 patients with diabetes, since high glucose levels would increase microvascular permeability and oedema, and facilitate the transendothelial migration of capillary neutrophils to alveolar air spaces (225) (Figure 7). It is not yet unclear, however, whether it is the hyperglycaemia *per se*, or other features associated with T2DM, that induces this response. Pulmonary NETs may also promote the oxidation of SARS-CoV-2 due to the generation of reactive oxygen species by NAD(P)H oxidases during NET formation (228). Oxidised viruses could activate TLR4 that is highly expressed in inflammatory immune cells, and subsequently enhance the TLR4-mediated inflammatory response and cytokine storm (234).

### Endothelial Dysfunction, Diabetes, and COVID-19

Given the route of entry of the SARS-CoV-2 virus into the body via the lungs, together with the primary symptoms that can accrue in terms of pulmonary oedema, inflammation and pneumonia, it is clear that the first-line cellular contact for the virus constitutes alveolar type 1 and type 2 epithelial cells that line the respiratory tract from nose to alveoli, together with the resident macrophages in the lungs, which help to raise the alarm for other inflammatory and immune cells to drive a defensive reaction (Figure 7). However, early in the pandemic, it was noted that cardiovascular complications were also widely experienced in severe cases of COVID-19, with symptoms associated with thrombosis and coagulation most prominent, increasing the risk of deep vein thrombosis, heart attack, stroke and peripheral ischaemia, often presenting in the form of “COVID toe” (235–237).

The endothelium was quickly identified as central to the vascular effects associated with COVID-19, not only because of the intimate proximity of alveolar epithelial and endothelial cells to facilitate efficient gas exchange, but also because endothelial cells express ACE2 and are highly regulated by many of the mediators that are modulated by changes in ACE2 presence or activity. An early case study involving post-mortem tissue suggested that SARS-CoV-2 can be found in endothelial cells, not only in the lungs (238) but also in other organs, and that inflammation and occlusion of small blood vessels was also evident in these patients (239).

Endothelial dysfunction is a feature of diabetes, driven by a range of mediating processes, including oxidative stress and inflammation (Figure 7). Indeed, endothelial dysfunction is considered to be the prime cause of accelerated atherogenesis in diabetes, leading to many of the secondary conditions, including coronary artery disease, ischaemic stroke, peripheral vascular disease (contributing to neuropathy and foot ulcers) and nephropathy (240). Hyperglycaemia-induced oxidative stress is a likely mechanism underpinning much of the endothelial dysfunction associated with diabetes (241), but elevated inflammation, resulting in enhanced expression of adhesion molecules [VCAM-1, ICAM-1, P-selectin (242–245)], is also considered to be key. Given that the endothelium in patients with diabetes is already compromised via these processes, it is perhaps inevitable that the impact of further endothelial dysfunction driven by COVID-19 might be more profound in this patient group, particularly if compounded by the effects of old age. What is not yet clear is the relative importance of changes in endothelial function in the lung to drive systemic inflammation, either through ensuing viraemia, extensive inflammation, or both.

### Possible Drivers of Endothelial Dysfunction in COVID-19

Endothelial cells are uniquely positioned at the interface between circulating blood and underlying tissues. The key role of the endothelium as the local modulator of vascular tone in response to a wide range of local physical, chemical and inflammatory mediators, necessitates a vast array of sensors and receptors which combine to generate an integrated, outgoing range of signals that in turn influence vascular smooth muscle contraction, proliferation, permeability, angiogenesis, thrombosis and inflammation (246, 247). Prime amongst these mediators is NO (248), but PGI_2_ is another important protective agent (249, 250), acting in opposition to the contracting factors that include angiotensin-II (251), thromboxane A_2_ (TXA_2_) (252) and endothelin-1 (ET-1) (253). These mediators have influence over other processes that could be important in either moderating or driving severe symptoms associated with COVID-19, both directly related to cardiovascular function and indirectly associated with acute and chronic respiratory symptoms. Some of the key mediators will be considered in turn, with a particular focus on possible interplay between diabetes and COVID-19.

### Nitric Oxide

NO is a key indicator of endothelial function and its wide-ranging actions ensure that it is critical in determining vascular health. NO is synthesised *de novo* on demand and its free radical characteristics ensure that its effects are confined to the locality of its generation and are short-lived [reviewed by (254)]. The level of stimulation is determined by an integrated Ca^2+^ signal derived from multiple inputs, including sheer stress (255), hypoxia (256), and several endothelium-dependent vasodilators [e.g., bradykinin, angiotensin-(1–7), reviewed in (257)]. The reaction with superoxide (^.^${\text{O}}_{2}^{-}$), is particularly important because it not only inactivates NO, but also generates a highly oxidising, cytotoxic product in the form of peroxynitrite (ONOO^−^) [reviewed by (258)].

A healthy endothelium promotes vasodilatation, vascular permeability and controlled angiogenesis, while at the same time inhibiting vascular smooth muscle cell proliferation, platelet adhesion and aggregation, and inflammatory cell adhesion (240). The cardiovascular impact of diabetes is at least partly due to loss of NO on account of both reduced synthesis and increased inactivation by superoxide (259, 260). The role of NO in COVID-19-induced changes in vascular function that could contribute to severe symptoms in the acute phase of COVID-19 are currently unknown. Certainly, reduced capacity for pulmonary dilatation, together with increased adhesion and infiltration of inflammatory cells and the additional risk of microthrombi precipitated by reduced NO, would all contribute to poor lung function and exacerbated inflammation. However, reduced NO production would also lead to reduced risk of vascular endothelial growth factor (VEGF)-induced hyperpermeability and angiogenesis, which is counter to the clinical findings (42, 238). Likewise, VEGF is increased in diabetes (261), although the profile of expression varies between T1DM and T2DM (262).

It is worth noting, however, that the contribution of eNOS to overall NO can be relatively small under pathological conditions, with high concentrations generated from the inducible isoform of the enzyme (iNOS) in activated macrophages and activated neutrophils (263), both of which are associated with COVID-19 (42). NO generated from inflammatory cells can act to destroy invading pathogens and indeed, iNOS-derived NO is considered critical in driving vascular collapse in animal models of sepsis, although the role in complications associated with sepsis in humans is less clear-cut (264). Until we fully understand the impact of COVID-19 on NO, it is difficult to determine whether inhibition of iNOS to reduce NO or delivery of inhaled NO (265) to alleviate symptoms driven by loss of eNOS-derived NO is advantageous. Clearly, if endothelium-associated microvascular dysfunction is critical in COVID-19, pre-existing diabetes will constitute a risk factor.

### Vascular Endothelial Growth Factor

Though VEGF-A is predominantly associated with angiogenesis, it also plays a significant role in the regulation of endothelial function [reviewed in: (266)] through, for example, the regulation of vascular permeability, or facilitating diapedesis of leukocytes (267). Expression of VEGF is controlled by hypoxia-induced expression of the transcription factor, HIF-1 (268), and it is not implausible to presume that both localised and systemic hypoxia caused by COVID-19 would lead to the overexpression or dysregulation of VEGF. The consequences of this would likely be an increase in vascular permeability and leakage, leading to increased oedema, as well as increased infiltration of leukocytes into surrounding tissues, further exacerbating the severe inflammatory symptoms of the infection. Angiogenesis is another symptom that has been found in patients who have died from COVID-19 (238) that is almost certain to involve VEGF.

### Oxidative Stress and Inflammation

The dual effect of loss of vasoprotective NO and the generation of cytotoxic, oxidising ONOO^−^ is considered to be important in the initiation and progression of atherogenesis (240, 269), not least in patients with diabetes (241). NO, together with PGI_2_ is not only a vital local mediator of vasodilatation, but also of inhibition of platelet aggregation. Diminution of this antithrombotic effect is exacerbated by increased oxidative stress in platelets of individuals with diabetes, which further inactivates NO in the target platelets themselves. Furthermore, evidence is accumulating to suggest that an imbalance in pro- (tissue plasminogen activator; t-PA) and anti- (plasminogen activator inhibitor-1; PAI-1) fibrinolytic factors in favour of PAI-1 in diabetes (270), could depress the fibrinolytic process that helps reverse coagulation associated with thrombus. Oxidative stress is also understood to be a driver of inflammation in endothelial cells, mediated by the expression of adhesion molecules on the surface, such as VCAM-1, ICAM-1 (240) and P-selectin [reviewed by (271–273)].

### Lipid Mediators of Inflammation

Eicosanoids are powerful lipid mediators of inflammation that are implicated in COVID-19. The role of eicosanoids in COVID-19 patients with diabetes is complex, as an imbalance in lipid mediator production is a feature of diabetes, contributing to the pathogenesis of the disease and associated complications [reviewed by (274, 275)]. Eicosanoids are lipid mediators synthesised by cyclo-oxygenase (COX), lipoxygenase (LOX) and P450 enzymes. While their impact in COVID-19 is currently poorly understood, SARS-CoV-1 can bind directly to the COX-2 promotor, increasing the expression of this inducible iso-enzyme, which is central to synthesis of prostanoids (276). In addition, the endoplasmic reticulum stress response, which can promote a cytokine storm, activates inositol-requiring enzyme 1[α] to upregulate COX-2 and prostaglandin E synthase, triggering the production of PGE_2_ amongst other prostaglandins (277). These findings suggest that an “eicosanoid storm” [reviewed by (278)] could contribute to the intense inflammatory response that accompanies severe COVID-19.

### Anti-inflammatory Drugs in COVID-19

There have been contradictory reports regarding the use of non-steroidal anti-inflammatory drugs (NSAIDs) to treat patients with COVID-19. NSAIDs include selective and non-selective inhibitors of the COX enzymes that catalyse the first committed step in synthesis of prostanoids, which contribute to pain and fever [reviewed in: (279)]. COX-1 is constitutive and central to synthesis TxA_2_ (amongst other prostanoid mediators) in platelets and endothelial cells, resulting in platelet aggregation and vasoconstriction. Aspirin is a weakly COX-1 selective inhibitor which is routinely used in primary prevention of myocardial infarction, except in patients with diabetes, where there is controversy over its benefits (280–284), perhaps suggesting eicosanoid imbalance in this patient group. By contrast, inducible COX-2 primarily synthesises PGI_2_, especially in microvascular endothelial cells, which opposes the actions of TxA_2_, resulting in inhibition of platelet aggregation and vasodilation (285, 286). The balance between PGI_2_ and TXA_2_ is critical for cardiovascular homeostasis (287, 288) and upsetting the balance can be detrimental. In addition, NSAIDs have differential and multifactorial effects on growth factor–induced angiogenesis and vascular permeability (289, 290) through inhibition of prostaglandins, particularly PGE_2_, which regulates vascular permeability (291) via the induction of VEGF and basic fibroblast growth factor (292). The anti-inflammatory effects of NSAIDs are broad: alongside the inhibition of prostaglandin synthesis, NSAIDs inhibit cytokine production, including IL-12 (293), IFN-γ (294), and IL-4 (295), thereby preventing T_h_1 and T_h_2-mediated responses. The use of NSAID may oppose the anti-inflammatory and pro-resolving roles of COX and ultimately lead to counter-intuitive prolongation of inflammation.

Advice early in the pandemic was to favour paracetamol over ibuprofen for COVID-19-related pain relief (296), due in part, to the observation that ibuprofen upregulates ACE2 in diabetic rats (297). However, this finding was not replicated in human clinical studies (298, 299) and this advice has since been withdrawn (300).

The corticosteroid, dexamethasone, promotes the production of pro-resolving mediators (301) and is now recommended for the treatment of the most severe symptoms in COVID-19 on account of reduced 28-day mortality amongst the treatment group of patients receiving invasive mechanical intervention or oxygen (302). However, the same trial found that there was a modest detrimental effect in patients not receiving respiratory support, again highlighting the importance of appropriate balance in the inflammatory response to COVID-19. In patients with diabetes, corticosteroids can have an impact on glycaemic control and other metabolic parameters and their use should be under careful clinical review (303).

### Thrombosis and Coagulopathy

An early clinical symptom identified in severe cases of COVID-19 was coagulopathy, with anywhere between 16–49% of patients admitted to ICU suffering thrombotic complications (304). The root cause(s) of thrombotic complications are not yet fully understood, but inflammatory cytokines have been implicated, along with endothelial dysfunction and stasis on account of immobility; this is not unique amongst severe infections. Post-mortem data from early casualties of COVID-19 indicated diffuse thrombosis throughout the lung microvasculature (305). Interestingly, while D-dimer levels are frequently raised in COVID-19 related coagulopathy, other coagulation cascade markers are not apparently consumed, as is seen with disseminated intravascular coagulation, perhaps pointing away from coagulation as the root cause and implicating platelet activation or dysfunctional fibrinolysis instead (306). Both of these possible causes would lead back to endothelial dysfunction as a crucial player in determining thrombotic potential, both through loss of anti-thrombotic NO and PGI_2_, and through a potential imbalance between endothelium-derived, fibrinolytic t-PA, and its countermeasure, PAI-1, in favour of the latter. With diabetes in mind as a potential primer for thrombosis in COVID-19, not only is diabetes associated with increased circulating inflammatory cytokines, but also with endothelial dysfunction, platelets in a hyperactive state, and an imbalance of t-PA and PAI-1 – all exacerbated by oxidative stress (307). The drive toward coagulopathy in COVID-19 is also likely to be boosted through enhanced generation of NETS, a feature that is common to diabetes (308) and infection (309).

Prophylactic and therapeutic uses of anticoagulant treatments are being investigated for COVID-19 patients (310). A retrospective study of 449 COVID-19 patients in China found no significant effect of heparin on 28-day mortality. However, there was a significant effect when only patients with a sepsis-induced coagulopathy score of >4 were analysed (311). Direct oral anticoagulants (DOACs) are also being investigated as potential COVID-19 treatments. There is evidence, from a study in ApoE^−/−^ mice, that DOACs may help to alleviate the endothelial dysfunction associated with diabetes (312), a potential confounding factor of COVID-19 coagulopathy in patients with diabetes. However, combining DOAC and antiviral treatments can lead to a sharp increase in plasma DOAC concentrations, which may increase the risk of haemorrhage in patients (313).

### COVID-19 Driven Diabetes-Like Syndrome

In children, the manifestation of β-islet autoimmunity is known to correlate with recent respiratory illness (314), including SARS-CoV-1 infection (315). In the case of SARS-CoV-2, there have been reports of COVID-19 patients exhibiting T1DM-like symptoms, despite no history of diabetes prior to hospitalisation. The first case study described a patient who presented with diabetes-like symptoms 1 month after they had been diagnosed with COVID-19, despite being normoglycaemic during their initial hospitalisation (316). Autoantibody production against β-islets was assessed, with the patient testing positive for glutamic acid decarboxylase-65 and negative for tyrosine phosphatase IA2 antibodies and zinc transporter 8 antibodies. As there was evidence of autoimmunity driving the development of diabetes, the patient was classified as having T1DM (316). Following this initial report, an increase in new-onset T1DM cases amongst children admitted to hospital with COVID-19 has been reported (68).

The induction of diabetes-like symptoms by SARS-CoV-1 infection was found in 39/520 SARS patients, who had no history of diabetes; only 2 patients were classified as having diabetes at 3 years post-hospitalisation (315). Diabetes was only assessed by glucose tolerance tests, with no measurement of autoantibodies; these type(s) of diabetes therefore cannot be classified. This study concluded that SARS-CoV-1 entered β-islets via ACE2 and the resultant damage caused an acute diabetes-like syndrome (315). Indeed, ACE2 is expressed throughout the pancreas, including in endocrine tissues and SARS-CoV-2 can infect both adult human pancreatic β cells and human pluripotent stem cell-derived pancreatic β cells (317).

Furthermore, a study in rats showed that an increase in angiotensin-II levels can result in an acute reduction in β-islet blood flow (318). This leads to the hypothesis that diabetes-like symptoms are caused by SARS-CoV-2 infection drive decreased ACE2 activity, which increases angiotensin-II and decreases β-islet function.

Therefore, the presence of diabetes-like symptoms in some patients after COVID-19 could be due to (i) induction of autoantibodies; (ii) direct infection of β-islets by SARS-CoV-2; or (iii) temporary β-islet function loss due to increased angiotensin-II. This is an evolving area of research and larger studies of “long-COVID,” the long-term effects of COVID-19, are now underway. We hypothesise a potential underlying mechanism of some of the long-COVID symptoms, particularly those of fatigue and general malaise, might be underlined by the dysregulation of metabolism and hyperglycaemia associated with a SARS-CoV-2 induced diabetes-like syndrome. There is some early evidence emerging to suggest that glucose control suffers perpetuated dysregulation at least in the acute phase after hospitalisation in hyperglycaemic patients with severe COVID-19 symptoms (319), although whether this is cause or effect is still to be deduced.

## Conclusions

COVID-19 is a disease with a wide array of possible outcomes, ranging from the benign right through to death. It soon became clear that age and pre-existing disease were two major risk factors for severe symptoms, and that diabetes was one of several disease profiles that was implicated as a risk factor. On the face of it, the potential links between diabetes and severe COVID-19 symptoms seem obscure, given that COVID-19 drives respiratory collapse, whereas diabetes is related to outcomes that are mediated by cardiovascular dysfunction, leading to retinopathy, neuropathy, peripheral vascular disease, stroke and myocardial infarction. Dig deeper, however, and parallels appear, not least with respect to dysfunction in various interconnecting systems and processes that could come together to drive a more severe package of symptoms in response to SARS-CoV-2 infection. Without doubt, subtle changes in the immune system and RAAS, together with inflammation, oxidative stress and endothelial dysfunction in diabetes have the potential to exaggerate the response triggered by SARS-CoV-2, ultimately driving one or more of the cellular processes that result in pulmonary thrombosis, increased vascular permeability and/or cytokine storm, resulting in respiratory failure.

Clearly, the story is a highly complex one: it must be remembered that the diabetes population is highly heterogeneous, with extremely diverse disease aetiology and severity, just as COVID-19 shows extreme diversity. Nevertheless, it is apparent that at least a sub-population of those with diabetes are less equipped to affect a quiet and efficient resolution of COVID-19, resulting instead in a monumental counter-regulatory failure with potentially fatal consequences. Additionally, evidence indicates that some individuals with COVID-19 develop a diabetes-like syndrome and it will be important to understand the mechanism by which this may occur. Current plausible explanations range through RAAS dysregulation, temporary β-islet destruction and induction of β-islet autoantibodies. More data on testing for β-islet autoantibodies and tracking the long-COVID symptoms are needed to begin to identify the underlying mechanisms behind these observations.

There is still a great deal to learn about COVID-19 before firm conclusions can be drawn as to the importance of the various potential players in determining the severity of the disease. The promise shown by the powerful anti-inflammatory agent, dexamethasone, which inhibits a multitude of pro-inflammatory pathways as a prophylactic agent or treatment for such symptoms, presents strong evidence for a central role for inflammation in symptom severity (302), but offers little insight to help predict what specific elements of the highly complex inflammatory system predispose to a poor outcome.

## Author Contributions

JR, AP, AT, AR, NB, PC, SM, JW, and IM were all involved in writing and editing the manuscript through several iterations. All authors contributed to the article and approved the submitted version.

## Conflict of Interest

The authors declare that the research was conducted in the absence of any commercial or financial relationships that could be construed as a potential conflict of interest.
